# Improving Fruit and Vegetable Consumption Among Low-Income Customers at Farmers Markets: Philly Food Bucks, Philadelphia, Pennsylvania, 2011

**DOI:** 10.5888/pcd10.120356

**Published:** 2013-10-03

**Authors:** Candace R. Young, Jennifer L. Aquilante, Sara Solomon, Lisa Colby, Mukethe A. Kawinzi, Nicky Uy, Giridhar Mallya

**Affiliations:** Author Affiliations: Jennifer L. Aquilante, Sara Solomon, Lisa Colby, Giridhar Mallya, Philadelphia Department of Public Health, Philadelphia, Pennsylvania; Mukethe A. Kawinzi, Nicky Uy, The Food Trust, Philadelphia, Pennsylvania.

## Abstract

**Introduction:**

We evaluated whether Philly Food Bucks, a bonus incentive program at farmers markets, is associated with increased fruit and vegetable consumption and Supplemental Nutrition Assistance Program (SNAP) sales at farmers markets in low-income areas.

**Methods:**

A convenience sample of 662 customers at 22 farmers markets in low-income neighborhoods in Philadelphia, Pennsylvania, was surveyed via face-to-face interviews. Questions addressed shopping characteristics, self-reported change in fruit and vegetable consumption, whether customers tried new fruits or vegetables, use of Philly Food Bucks, and demographic information. Market-level SNAP sales and Philly Food Bucks redemption data were also collected to monitor sales patterns.

**Results:**

Philly Food Bucks users were significantly more likely than nonusers to report increasing fruit and vegetable consumption (OR, 2.4; 95% CI, 1.6–3.7; *P* < .001) and to report trying new fruits or vegetables (OR 1.8; 95% CI, 1.2–2.7; *P* = .006). At the market level, average SNAP sales more than doubled at farmers markets in low-income areas in the first 2 years of the Philly Food Bucks program. At the city’s largest farmers market in a low-income area, the program was associated with an almost 5-fold higher increase in annual SNAP sales compared with baseline.

**Conclusion:**

Results from this study demonstrate that a bonus incentive program tied to SNAP was associated with self-reported increases in fruit and vegetable consumption and increased SNAP sales at participating farmers markets in low-income communities. More research is warranted to evaluate the long-term impact of bonus incentives on farmers market use, dietary behaviors, and health outcomes.

## Introduction

The local food environment is a determinant of food access and diet quality (1–3). This relationship is of particular concern in low-income urban communities where there are few supermarkets and an abundance of fast food outlets and corner stores. In Philadelphia, residents of low-income neighborhoods are half as likely to have access to grocery stores as residents of high-income neighborhoods (4).

Numerous strategies have been implemented to improve access to healthful foods in low-income communities, including supermarket financing initiatives (5), efforts to provide healthy foods at corner stores (6–8), and expansion of farmers markets (9,10). However, less attention has been devoted to improving affordability of healthful foods. During the past 4 decades, prices of healthful foods and beverages have increased relative to unhealthful items (11). Programs that increase the purchasing power for low-income consumers to buy healthful foods are promising approaches (9,12,13). However, the impact of bonus incentive programs has not been extensively evaluated.

Farmers markets may be particularly conducive for bonus incentive programs because they offer predominantly healthful foods (14,15) and increasingly are located in low-income communities and are equipped to accept Supplemental Nutrition Assistance Program (SNAP) benefits through the use of wireless technology (16,17). Philadelphia, Pennsylvania, is one of several cities and counties nationwide that have implemented bonus incentive programs targeting SNAP recipients as part of the Communities Putting Prevention to Work (CPPW) initiative of the Centers for Disease Control and Prevention, which aims to reduce chronic diseases through policy, systems, and environmental changes (18).

From 2010 to 2011, The Food Trust partnered with the Philadelphia Department of Public Health CPPW-funded Get Healthy Philly initiative to implement Philly Food Bucks, $2 bonus incentive coupons that could be redeemed only for fresh fruits and vegetables at farmers markets. The Philly Food Bucks program aimed to bring new customers to markets in low-income communities, to increase purchasing power for fruits and vegetables, to increase fruit and vegetable consumption among low-income customers, and to increase use of SNAP at farmers markets. Philly Food Bucks were distributed in 1 of 2 ways. They were distributed onsite at farmers markets along with SNAP purchases: a $2 bonus incentive coupon was received for every $5 in SNAP benefits used. (Original SNAP purchases did not need to be fresh fruits and vegetables to qualify.) Philly Food Bucks were also distributed by community organizations that serve SNAP-eligible populations to promote farmers market access among low-income residents. Those coupons could be redeemed without making a SNAP purchase.

Our study presents data from 2 sources: customer surveys and objective sales data from 22 farmers markets in low-income areas of Philadelphia. Ten of 22 markets were newly opened in 2010 to 2011 through Get Healthy Philly. The other 12 markets were in operation for 3 to 14 years. At schools near the 22 markets, more than 70% of students are eligible for free or reduced-price school meals (range, 72% to 95%), indicating that more than 70% of households near these markets are at or below 185% of the federal income poverty level (19,20).

This study addresses the following questions: What are the characteristics of Philly Food Bucks users? Among farmers market customers, are Philly Food Bucks users more likely than nonusers to report increased fruit and vegetable intake? What is the association between the Philly Food Bucks bonus incentive program and SNAP sales at farmers markets?

## Methods

The study protocol was determined to be exempt from review by the Institutional Review Board of the Philadelphia Department of Public Health.

### Data collection

#### Customer survey

A customer survey was conducted at farmers markets to assess self-reported dietary behaviors since visiting the market (change in fruit and vegetable consumption and trying new fruits and vegetables), shopping frequency, distance traveled to market, participation in SNAP and other food assistance programs in the past year, use of Philly Food Bucks, and demographic information.

Market staff conducted the 22-question survey in face-to-face interviews with a convenience sample of 662 shoppers at 22 farmers markets in low-income areas of Philadelphia from September to November 2011. Surveys were administered and recorded by 15 trained farmers market staff from The Food Trust. All interviewers were familiar with market operations and benefits accepted at the market (SNAP, Philly Food Bucks, vouchers for Special Supplemental Nutrition Program for Women, Infants, and Children [WIC], and vouchers for the Senior Farmers’ Market Nutrition Program). Market staff approached customers by asking them to participate in a brief survey about farmers markets. Refusals were not tracked but were estimated at 10%.

Surveys were collected during market hours, which varied by market. Survey collection was completed over the course of 1 to 5 market days, depending on size of market, volume of customers, and weather (eg, when it rained, an additional survey day was scheduled). Surveys took approximately 5 minutes to complete.

#### Market-level sales data

For the 22 markets in the study, SNAP and Philly Food Bucks data were tracked, including market-level SNAP sales (in dollars) for the 2009 through 2011 market seasons and Philly Food Bucks distribution and redemption (in dollars) for the 2010 and 2011 seasons. These data are used to reimburse farmers for SNAP and Philly Food Bucks purchases. Philly Food Bucks distribution and redemption are tracked by using the unique serial number on each coupon. In 2009, there were 12 markets in low-income neighborhoods; in 2010, 16; and in 2011, 22. The largest and longest-running market in a low-income community, based in West Philadelphia, accounted for 54% of our farmers market SNAP sales in 2011 and one-fourth of all farmers market SNAP sales in Pennsylvania. To examine differences in trends in SNAP sales before and after implementation of the Philly Food Bucks program, we focused on this site because sales data were available beginning in 2005. This market has been the subject of previous research (17) as a model and case study for markets in other urban, low-income areas.

#### SNAP eligibility data

The Pennsylvania Department of Public Welfare publishes monthly data on the SNAP-eligible population of Philadelphia County (21). Monthly data on the percentage of the Philadelphia population eligible for SNAP were averaged for May through November for each year from 2005 through 2011 to serve as a control variable in examining increases in SNAP sales.

### Statistical analysis

#### Customer survey

Customer surveys were scanned using Remark Office OMR 7.0 (Gravic Inc, Malvern, Pennsylvania) and data analyzed in SPSS Version 17.0 (IBM Corporation, Chicago, Illinois). Cross tabulations and χ^2^ statistics were calculated to analyze demographic and shopping characteristics of Philly Food Bucks users and whether they differed significantly from non–Philly Food Bucks users.

Multivariate binary logistic regression was used to examine predictors of self-reported increased fruit and vegetable consumption and trying new fruits or vegetables since becoming a market customer. Increased fruit and vegetable consumption was assessed by using the following survey item: “Since becoming a customer at this market, do you eat more, less, or the same amount of fruits and vegetables?” and coded as a dichotomous dependent variable (increased vs decreased and no change). Trying new fruits or vegetables was measured by using the following survey item: “Since becoming a customer at this market, have you tried any new or unfamiliar fruits or vegetables?” (tried new vs no).

Explanatory variables included in full models were having used Philly Food Bucks at the market (yes vs no) and characteristics significantly associated with Philly Food Bucks use: receiving nutrition information at market (yes vs no), becoming a customer during the 2011 season (yes vs no), walking or biking to market (yes vs no), and demographic variables sex (female vs male), age (18–25 vs 26 or older) and race (African American, Hispanic, or Asian vs white). Subjects missing data for 1 or more variables in a model were dropped from analysis in that model.

A Wald statistic with a *P* value of .05 or less was considered significant. Odds ratios (ORs) and 95% confidence intervals (CIs) for odds ratios were calculated by exponentiating e^β^, where β equals the parameter estimate for each explanatory variable (22).

#### Market-level sales data

Given the increase in the number of markets in low-income areas able to accept SNAP over time, we recorded the total SNAP sales and average SNAP sales per market for each year. Similarly, total Philly Food Bucks redemption and average Philly Food Bucks redemption per market were tracked and calculated for 2010 and 2011. Bivariate linear regression was used to estimate how market-level Philly Food Bucks redemption (in dollars) predicted SNAP sales (in dollars) in 2010 and 2011.

In addition, by using sales data from the largest and longest operating market in a low-income community in Philadelphia, we assessed how trends in total annual SNAP sales differed between the pre–Philly Food Bucks period (2005–2009) and the Philly Food Bucks implementation period (2009–2011). Linear regressions were run for the 2 periods, adjusting for the annual percentage of the Philadelphia population eligible for SNAP. *P* values and 95% CIs for the slopes were compared with rates of growth in SNAP sales before and during the Philly Food Bucks program.

## Results

### Customer survey

In total, 662 shoppers completed surveys at the 22 markets in low-income communities. A mean of 30 surveys were collected per market (range, 18–40). One hundred seventy-five (27%) respondents were Philly Food Bucks users, 433 (65%) were not Philly Food Bucks users (Table 1); 54 (8%) respondents were excluded because use of Philly Food Bucks was unknown.

**Table 1:** Characteristics of Farmers Market Customers, by Philly Food Bucks Use,^a^ at 22 Farmers Markets in Low-Income Communities, Philadelphia, Pennsylvania, 2011

Characteristic	Philly Food Bucks Users (n = 175), %	Non–Philly Food Bucks Users (n = 433), %
**Sex**		
Female	75.9	71.5
**Race/ethnicity** b		
African American	52.0	45.3
White	22.3	42.8
Hispanic	14.0	6.5
Asian	7.0	2.1
Other	4.7	3.3
**Age, y**		
18–25	12.4	14.4
26–40	36.1	29.6
41–65	38.5	35.7
≥65	13.0	20.3
**Shopping characteristics**		
Began shopping at market in 2011^b^	50.9	34.5
Walk or bike to market^b^	67.8	54.4
Received nutrition education while at market^b^	84.6	55.8
Increased fruit and vegetable consumption^b^	71.1	46.3
Tried new fruits or vegetables^b^	56.7	41.3
Visit the market every week	45.3	41.5
Travel 3 blocks or less to get to the market	34.5	35.2
Report that prices at market are less expensive than food stores in neighborhood	43.9	37.2
Have used a SNAP card at the farmers market^b^	72.4	8.8

Abbreviation: SNAP, Supplemental Nutrition Assistance Program.

a Sample size = 608; Philly Food Bucks use was unknown for 54 (8%) respondents, which are excluded from the table.

b χ^2^ test showed significant association (*P* ≤ .005).

Among Philly Food Bucks users, 72% had made a SNAP purchase at market, compared with 9% of non–Philly Food Bucks users (*P* < .001). Compared with nonusers, Philly Food Bucks users were more likely to be nonwhite, to be a new customer at the market in 2011, to walk or bike to market, and to report receiving nutrition education at market. There were no significant differences between those who did and did not use Philly Food Bucks and frequenting the market on a weekly basis, traveling 3 blocks or less to get to market, or reporting prices at market were less expensive than local food stores.

In logistic regression models adjusting for other factors, Philly Food Bucks users were significantly more likely than nonusers to report eating more fruits and vegetables since becoming a market customer (OR, 2.4; 95% CI, 1.6–3.7; *P* < .001) and to report trying new or unfamiliar fruits or vegetables since becoming a market customer (OR, 1.8; 95% CI, 1.2–2.7; *P* = .006) (Table 2).

**Table 2:** Multiple Logistic Regression for Factors Associated With Increasing Fruit and Vegetable Consumption and Trying New Fruits or Vegetables Among Customers at Farmers Markets in Low-Income Communities, Philadelphia, Pennsylvania, 2011

Factors in Model	Model 1: Increasing Fruit and Vegetable Consumption (n = 531; missing = 131)		Model 2: Trying New Fruits or Vegetables (n = 538; missing = 124)	
	OR (95% CI)	*P* Value^a^	OR (95% CI)	*P* Value^a^
Used Philly Food Bucks	2.4 (1.6–3.7)	<.001	1.8 (1.2–2.7)	.006
Received nutrition education at market	1.8 (1.2–2.7)	.003	1.5 (1.0–2.2)	.04
Walked or biked to market	1.0 (0.7–1.4)	.98	1.4 (1.0–2.1)	.05
New customer at market in 2011	1.1 (0.8–1.6)	.59	1.0 (0.7–1.4)	.91
Female	0.9 (0.6–1.3)	.60	1.0 (0.7–1.5)	.83
Aged 18 to 25 years	1.8 (1.1–3.0)	.03	0.8 (0.5–1.3)	.41
African American, Hispanic, or Asian	0.8 (0.5–1.1)	.17	0.7 (0.5–1.0)	.03

Abbreviation: CI, confidence interval.

a Wald statistic.

Participating in Philly Food Bucks, receiving nutrition education at market, and younger age (18 to 25 years) were significantly and positively associated with reporting an increase in fruit and vegetable consumption (Table 2). No significant associations were noted in the model between increased fruit and vegetable consumption and walking or biking to market, being a new customer at the market, sex, or race/ethnicity.

Participating in Philly Food Bucks, receiving nutrition education at market, and walking or biking to market were significantly and positively associated with trying new fruits or vegetables. Race/ethnicity other than white was negatively associated with the likelihood of trying new fruits or vegetables (Table 2). No significant associations were noted in the model between trying new fruits or vegetables and being a new customer at the market, sex, or age.

### Market sales

SNAP transactions and sales per market increased steadily during the Philly Food Bucks intervention (Table 3). SNAP sales at farmers markets in low-income areas increased by more than 300% from $12,431 in 2009, before Philly Food Bucks, to $52,405 in 2011, after 2 years of the Philly Food Bucks program. Although the number of markets that accepted SNAP also increased during the same period, average SNAP sales per market more than doubled from $1,036 in 2009 to $2,382 in 2011.

**Table 3:** SNAP and Philly Food Bucks Sales at Farmers Markets in Low-Income Communities, Philadelphia, Pennsylvania, 2009 to 2011

Characteristic	SNAP			Philly Food Bucks^a^		
	2009	2010	2011	2009	2010	2011
No. of participating markets	12	16	22	0	16	22
Total sales, $	12,431	25,032	52,405	NA	10,856	25,914
Average sales per market, $	1,036	1,565	2,382	NA	679	1,178
Increase from previous year in average sales per market, %	35.4.	51.0	52.3	NA	NA	73.5

Abbreviations: SNAP, Supplemental Nutrition Assistance Program; NA, not applicable

a Philly Food Bucks distributed onsite at farmers markets through SNAP purchases accounted for 75% of redemptions. Philly Food Bucks distributed through community organizations accounted for 25% of redemptions.

Average Philly Food Bucks redemptions per market almost doubled from $679 per market in 2010 to $1,178 per market in 2011. Philly Food Bucks distributed at farmers markets and tied to SNAP purchases ($2 in Philly Food Bucks for every $5 in SNAP purchases) accounted for 75% of Philly Food Bucks redeemed. Philly Food Bucks distributed by community organizations accounted for 25% of redemptions.

Market-level SNAP and Philly Food Bucks sales were highly correlated for the 22 markets in the study. Bivariate linear regression models found that every $1 in Philly Food Bucks sales was associated with $3.75 in SNAP sales in 2010 and $2.74 in SNAP sales in 2011 (versus the bonus incentive model of $2 for $5, or $1 in Philly Food Bucks for every $2.50 of SNAP sales).

By examining data from 2005 to 2011 for the largest and longest-operating market in the city, linear regression analyses controlling for SNAP eligibility revealed that total annual SNAP redemption increased during the Philly Food Bucks period (slope, 2.01; 95% CI, 0.91–3.11) at nearly 5 times the rate as the pre–Philly Food Bucks period (slope, 0.41; 95% CI, 0.06–0.76).

## Discussion

This evaluation lends support to the effectiveness of bonus incentive programs in improving nutrition behaviors of low-income residents. Our results suggest that among customers of farmers markets in low-income communities, participants in the Philly Food Bucks program were significantly more likely than nonparticipants to report eating more fruits and vegetables and trying new fruits or vegetables since becoming customers at the market. Markets participating in the Philly Food Bucks program demonstrated larger increases in SNAP sales per market than were observed before the bonus incentive program. Associations between Philly Food Bucks and SNAP sales were stronger than expected given the $2 for $5 bonus incentive model (or $1 in Philly Food Bucks for every $2.50 of SNAP sales).

The findings related to increased consumption of fruits and vegetables are consistent with those of prior studies assessing bonus incentive programs (12,13); however, there are key differences in the type and size of the incentives offered, as well as study settings and designs. Anderson and colleagues (12) found that women participating in WIC in Genesee County, Michigan, increased their fruit and vegetable consumption from baseline when provided one $20 farmers market coupon during a 5-month period. The effect was even greater for those receiving a brief, interactive nutrition education session. In another study, Herman et al (13) demonstrated that women participating in WIC in Los Angeles, California, purchased a wide variety of fruits and vegetables when given $40 per month in vouchers during 6 months for use in supermarkets and farmers markets.

In our study, participants were customers of farmers markets in low-income neighborhoods in Philadelphia. SNAP recipients received coupons based on SNAP purchases at farmers markets or through community organizations with a high percentage of SNAP-eligible clients. In contrast to studies cited above, in our study the size of the incentive was not fixed. Overall, our study population was neither exclusively female nor exclusively comprised of WIC participants. Our study also had a higher percentage of African Americans and a higher mean age compared with other studies. These characteristics may enhance the generalizability of our study to other large urban areas.

Assessment of increased fruit and vegetable consumption in our study was based on self-report at a single point in time. We also explored SNAP redemption levels at farmers markets before and during the Philly Food Bucks intervention. The growth in SNAP sales attributable to Philly Food Bucks may indicate that the incentive bolstered farmers market use among SNAP recipients. The bonus incentive program could have attracted low-income customers to markets when they otherwise would not have patronized them or it could have prompted them to “spend more to earn more.” Other factors also likely contributed to increased SNAP sales, including temporal increases in SNAP eligibility and farmers market use citywide, expansion of farmers markets in low-income neighborhoods, community-based promotion of markets, and nutrition education offered at markets.

Our findings are subject to several limitations. First, the study involved a convenience sample, which may not accurately reflect a representative sample of low-income residents who did or did not use Philly Food Bucks at farmers markets. Second, Philly Food Bucks participation was not randomly assigned, so differences in fruit and vegetable consumption may reflect unmeasured confounders or that customers who want to increase fruit and vegetable consumption are more likely to participate in the Philly Food Bucks program. Third, the measure of fruit and vegetable consumption was self-reported, based on 1 survey question, and cross-sectional in nature, limiting causative conclusions. Social desirability bias may have led to over-reporting of increased fruit and vegetable consumption. However, this measure was supplemented by self-report of trying new fruits and vegetables, which was also positively associated with Philly Food Bucks use. Fourth, there could be a seasonal effect on our findings because the customer survey was conducted toward the end of the farmers market season. Fifth, increases in SNAP sales beyond baseline could be attributable to Philly Food Bucks and other factors, such as increases in the SNAP-eligible population that were accounted for in our analyses or increases in food prices at our farmers markets that were not accounted for in our analyses. Lastly, these findings may not be generalizable to rural communities.

Despite these limitations, the initiation of the Philly Food Bucks program was associated with a significantly higher likelihood of self-reported increases in fruit and vegetable consumption and trying new fruits and vegetables in addition to increased per-market SNAP sales at farmers markets in low-income communities. This pilot program highlights the potential health impact of bonus incentive programs for low-income populations.

Future work can build on successes of bonus incentive programs at farmers markets. Pilot programs, such as the US Department of Agriculture’s Healthy Incentives Pilot in Massachusetts, will explore how integrating bonus incentives into the Electronic Benefits Transfer system can further increase fruit and vegetable consumption and SNAP use among low-income urban populations (23). Public and private organizations should prioritize investing in food access initiatives that address affordability as well as availability. Federal food programs should fund and further evaluate bonus incentive programs to decrease barriers to fruit and vegetable consumption among low-income populations.
